# Assessing Consistency of Drug-Drug Interaction-Related Information Across Various Drug Information Resources

**DOI:** 10.7759/cureus.13766

**Published:** 2021-03-08

**Authors:** Atiqulla Shariff, Sathvik Belagodu Sridhar, Neelu Farhath Abdullah Basha, Shamma Sulaiman Hasan Bin Taleth Alshemeil, Noora Adel Ahmed Aljallaf Alzaabi

**Affiliations:** 1 Department of Clinical Pharmacy & Pharmacology, Ras Al Khaimah College of Pharmaceutical Sciences, Ras Al Khaimah Medical and Health Sciences University, Ras Al Khaimah, ARE

**Keywords:** drug information resources, drug-drug interactions, interacting drug pairs, severity, clinical effects, mechanism, management, consistency, scope score, completeness score

## Abstract

Background

Information related to drug-drug interactions (DDIs) varies significantly from one drug information (DI) resource to another. These variations pose challenges for healthcare professionals in making the right decisions regarding using some of the drug combinations in needy patients. The objective of this study was to review eight different DI resources for scope, completeness, and consistency of information related to DDIs.

Methodology

A total of eight DI resources, namely, Micromedex^®^, Portable Electronic Physician Information Database^©^, UpToDate^®^, Medscape.com drug interaction checker, Drugs.com drug interaction checker, Stockley’s Drug Interactions (ninth edition, 2010), Drug Interactions Analysis & Management: Facts and Comparisons 2014 (ninth edition, 2014), and the drug interaction appendix of the British National Formulary-76, were compared. Each DI resource was scored for scope by calculating the percentage of interactions that had an entry in each resource. A completeness score was calculated for each resource describing severity, clinical effects, mechanism, and DDI management. The consistency of the information was assessed using Fleiss Kappa (k) score estimated using ReCal3 0.1 (alpha) web service and Statistical Package for the Social Sciences version 24.

Results

The scope score was the highest (100%) for UpToDate^®^ and Portable Electronic Physician Information Database^©^, whereas the completeness score was the highest (100%) for Drug Interaction Analysis & Management: Facts and comparisons 2014. The inter-source reliability scores among the eight different DI sources were poor (k < 0.20, *p *< 0.05) for documentation of information related to severity, clinical effects, mechanism, and management of DDIs.

Conclusions

Variations in the information cause uncertainty among healthcare professionals concerning interacting drug pairs in clinical practice. This may also increase the possibility of adverse drug outcomes when interacting drug pairs are used in at-risk patients. We recommend comprehensive preventive and management strategies for DDIs depending on a uniform scale of severity and clinical effects across various DI resources.

## Introduction

Multiple drug therapy is often evident in clinical practice. Combination drug therapy is beneficial; however, it also carries the risk of drug interactions [1,2]. Drug interactions are one of the significant causes of drug-related problems. Drug interactions contribute to approximately 3-26% of all adverse drug events leading to hospital admissions [3,4]. Drug interactions are known to increase the length of hospital stay and increase healthcare costs [5]. Although polypharmacy is one of the preventable causes of drug interactions, it remains impossible to reduce the number of drugs taken by many patients [6]. Therefore, having a significant drug interaction screening program in place can prevent drug interactions and related adverse drug outcomes [7].

Several databases and resources provide information on the type, mechanism, severity, and pharmacological consequences of drug interactions. Some of these are ready reference resources (British National Formulary), while others are specific detailed drug interaction texts (Stockley’s Drug Interactions). In addition, there are specific online/web-based tools that need to be subscribed to (Micromedex®), some of which are freely accessible (Drugs.com drug interaction checker) [7,8]. The information related to drug interactions varies significantly from one resource to another [8,9].

The variation in information among different databases and resources ranges widely regarding the presence or absence of interaction to the different levels, severity, mechanism(s), clinical relevance, and management of drug interactions [9,10]. These wide variations in information pose challenges for clinical pharmacists and other healthcare professionals in making the right decision regarding the use of some of the combinations in needy patients, especially those who are at risk of developing drug interactions [11]. Therefore, selecting the right drug information (DI) resource is essential while performing a comprehensive medication review to identify and resolve drug interactions [12]. The objective of this study was to review eight different DI resources for scope, completeness, and consistency of information related to drug-drug interactions (DDIs). This article was previously presented as a conference poster at the Dubai International Pharmaceutical and Technologies Conference and Exhibition (DUPHAT) on February 25-27, 2020.

## Materials and methods

Study design and study site

This was a cross-sectional systematic comparative study conducted at the Drug Information Centre, Department of Clinical Pharmacy & Pharmacology, Ras Al Khaimah College of Pharmaceutical Sciences (RAKCOPS), Ras Al Khaimah Medical & Health Sciences University (RAKMHSU), Ras Al Khaimah, United Arab Emirates (UAE). Approval for conducting the study was obtained from the institutional research and ethics committee (REC no. RAKMHSU REC-132-2019-UG-P).

Selection of drug pairs

A vetting committee was constituted to select the most common drug pairs used in the UAE. The vetting committee consisted of two doctors from the internal medicine department, a professor from the clinical pharmacy department, and a pharmacist from the community practice and hospital practice. Based on the consensus, a list of 50 most commonly used drug pairs was prepared.

Selection of drug information resources

A total of eight DI resources, namely, Micromedex® [13], Portable Electronic Physician Information Database (PEPID^©^) [14], UpToDate® [15], Medscape.com drug interaction checker (MDIC) [16], Drugs.com drug interaction checker (DDIC) [17], Stockley’s Drug Interactions, ninth edition, 2010 (SDI-2010) [18], Drug Interactions Analysis & Management: Facts and comparisons 2014, ninth edition, 2014 (DIAM-2014) [19], and the drug interaction appendix of the British National Formulary (BNF-76) [20] were compared. We selected these DI resources as health professionals in UAE hospitals and pharmacies most commonly use them to obtain information regarding drug interactions.

Scope score calculation

Each DI resource was scored for scope by calculating the number of drug pairs that had an entry in each resource. For scoring, the drug pairs were characterized into three categories, namely, interacting drug pairs (drug pairs with documented interaction in the given DI resource), non-interacting drug pairs (drug pairs with no interaction documented in the given DI resource), and not-listed drug pairs (drug pairs which did not have an entry in the given DI resource). Both interacting and non-interacting drug pairs were given a score of one, whereas drug pairs under the not-listed category were given a score of zero [12].

For example, in SDI-2010, out of the 50 drug pairs analyzed, we found 43 interacting drug pairs, four non-interacting drug pairs, and three not-listed drug pairs. Accordingly, the overall scope score for SDI-2010 was 47 (43 + 4 = 47). Further, the percentage scope score was calculated by dividing the raw score by the total number of drug pairs analyzed and multiplying the result with 100 (47/50 × 100 = 94% for SDI-2010). We considered interaction with the greater value of classification for the drug pairs with more than one entry of interaction with different degrees of risk, severity, or documentation in any DI resource.

Completeness score calculation

Four components, namely, clinical effects (or outcome), severity, mechanism, and management (or course of action) of the identified drug interactions, were assessed to calculate the completeness score. Each interacting drug pair was given a score of one for each of the components documented, and the overall completeness score was calculated by adding the individual scores and dividing it by the total number of interacting drug pairs in that particular DI resource [12]. For example, out of the 43 interacting drug pairs in SDI-2010, severity and clinical effects were mentioned for all the 43 drug pairs, whereas mechanism and management were mentioned only for 33 and 38 interacting drug pairs, respectively. Accordingly, the overall completeness score for SDI-2010 was 3.65 (43 + 43 + 33 + 38 = 157/43 = 3.65). The overall percentage completeness score was calculated by dividing the overall completeness score by four (total number of components assessed) and multiplying the answer with 100 (3.65/4 × 100 = 91.25% for SDI-2010).

Assessment of the consistency of information

The authors assessed the consistency of information related to all the four aspects of DDIs for all the 50 drug pairs. Different DI resources provide different scales regarding the severity of the drug interactions. Therefore, we developed and validated a uniform severity rating scale (Table 1, Appendix). Severity scores were then standardized for individual DI resources before assessing consistency.

**Table 1:** Standardized severity rating scale. PEPID^©^: Portable Electronic Physician Information Database; MDIC: Medscape.com Drug Interaction Checker; DDIC: Drugs.com Drug Interaction Checker; SDI-2010: Stockley’s Drug Interactions, ninth edition, 2010; DIAM-2014: Drug Interactions Analysis & Management-facts and comparisons, ninth edition, 2014; BNF-76: British National Formulary, 76th edition

Drug Information Resources								Standardized Severity Category
Micromedex^®^	PEPID^©^	UpToDate^®^	MDIC	DDIC	SDI-2010	DIAM-2014	BNF-76	
Minor	Non-significant, minor	Minor	Minor	Minor	Non-significant	Minor	Mild	Mild
Moderate	Moderate	Moderate	Significant: monitor closely	Moderate	Consider monitoring	Moderate	Moderate	Moderate
Major, contraindicated	Significant, life-threatening	Major	Serious-use alternative, contraindicated	Major	Life-threatening: contraindicated, Significant hazard	Major, avoid: contraindicated	Severe	Major

Similarly, to ease the consistency assessment of the other three components (clinical effects, mechanism, and management of DDIs), another numerical score scale (Table 2, Appendix) was developed and validated for coding or scoring the gathered information.

**Table 2:** Numerical score scale coding for assessing the consistency of information on DDI. DDI: drug-drug interaction; DI: drug information

Score or code	Component of DDI			
	Severity	Clinical effect	Mechanism	Management
0	For the drug pairs that were not listed in the DI resource			
1	For the drug pairs that were mentioned as non-interacting in the DI resource			
2	Mild	Not applicable		
3	Moderate			
4	Major			
5-12	Not applicable	If the information in all the eight DI resources was different from one another		
13	If the DI resource does not provide any information (not mentioned) about the severity or clinical effects or mechanism or management of interacting drug pairs			
Identical numerical score	Not applicable	If the information is identical in more than one drug information resource, all the DI resources with identical information were given the same numerical score		

Authors individually collected and recorded the information related to all the 50 drug pairs. The information given in the DI resources was captured in excel sheets without any inputs by the authors. The collected data were then coded or scored according to Tables 1, 2. Further, investigators met every week to discuss and ascertain that the collected data were precise. Discrepancies, if any, were resolved based on consensus. Additionally, the consistency of the recorded information among the study investigators was also assessed.

Finally, the consistency among the DI resources was assessed only after confirming the consistency among the study investigators in recording the data was satisfactory, as indicated with Fleiss Kappa (k) score of 1 (very good). Fleiss Kappa (k) score was estimated using ReCal3 0.1 (alpha) web service and SPSS (version 24.0, IBM, Armonk, New York, USA) to assess the consistency of the recorded information among the investigators as well as among the different DI resources [21,22].

## Results

The vetting committee selected a total of 50 most commonly used drug pairs in the UAE. The complete list of drug pairs is given in Table 3.

**Table 3:** List of drug pairs studied. Co-trimoxazole: trimethoprim + sulfamethoxazole; HRT: estrogens (conjugated/equine) and medroxyprogesterone; hormonal contraceptives: estrogens (conjugated/equine) and medroxyprogesterone

Drug pairs		Drug pairs	
Captopril +	Digoxin	Nimodipine +	Carbamazepine
Aciclovir +	Cimetidine	Amlodipine +	Simvastatin
Albendazole +	Carbamazepine	Carbamazepine +	Clopidogrel
Allopurinol +	Insulin	Cefpodoxime +	Pantoprazole
Prazosin +	Amlodipine	Chloramphenicol +	Vitamin B12
Gentamicin +	Vancomycin	Clopidogrel +	Cimetidine
Amiodarone +	Eplerenone	Clopidogrel +	Atorvastatin
Telmisartan +	Glibenclamide (Glyburide)	Hormonal contraceptives +	Metronidazole
Losartan +	Aspirin	Prednisolone +	Pneumococcal vaccine
Metformin +	Aspirin	Co-trimoxazole +	Phenytoin
Metformin +	Furosemide	Digoxin +	Atorvastatin
Metformin +	Hydrochlorothiazide	Bromocriptine +	Pseudoephedrine
Metformin +	HRT	Folic acid +	Methotrexate
Metformin +	Warfarin	Fusidic acid +	Atorvastatin
Ebastine +	Ketoconazole	Gold sodium thiomalate +	Penicillamine
Olanzapine +	Quetiapine	Heparin +	Glyceryl trinitrate
Thioridazine +	Duloxetine	HRT +	Warfarin
Prochlorperazine +	Levodopa	Ferrous sulfate +	Levothyroxine
Haloperidol +	Phenytoin	Ferrous sulfate +	Methyldopa
Azathioprine +	Sulfasalazine	Clarithromycin +	Esomeprazole
Azathioprine +	Pneumococcal vaccine	Methotrexate +	Omeprazole
Fluconazole +	Hormonal contraceptives	Glyceryl trinitrate +	Sildenafil
Ketoconazole +	Omeprazole	Methadone +	Ciprofloxacin
Ketoconazole +	Simvastatin	Tramadol +	Citalopram
Alendronate +	Ibuprofen	Atorvastatin +	Warfarin

The percentage scope score was the highest (100%) for PEPID^©^ and UpToDate® when compared to SDI-2010 and BNF-76, which was 94%. The detailed scope scores are presented in Table 4.

**Table 4:** Scope score for the DI resources studied. PEPID^©^: Portable Electronic Physician Information Database; MDIC: Medscape.com Drug Interaction Checker; DDIC: Drugs.com Drug Interaction Checker; SDI-2010: Stockley’s Drug Interactions, ninth edition, 2010; DIAM-2014: Drug Interactions Analysis & Management-facts and comparisons 2014, ninth edition, 2014; BNF-76: British National Formulary, 76th edition; DI, drug information

Drug information resource	No. of interacting drug pairs (X)	No. of non-interacting drug pairs (Y)	No. of drug pairs not-listed	Total [n = 50]	Scope score (X+Y)	% scope score (X+Y/50)×100
Micromedex^®^	27	21	02	50	48	96
PEPID^©^	44	06	00	50	50	100
UpToDate^®^	39	11	00	50	50	100
MDIC	35	13	02	50	48	96
DDIC	41	07	02	50	48	96
SDI-2010	43	04	03	50	47	94
DIAM-2014	17	32	01	50	49	98
BNF-76	21	26	03	50	47	94

The completeness score for clinical effects was 100% for all the DI resources. The completeness score for severity was 100% for all the DI resources except for BNF-76 (76.1%). Micromedex® and DIAM-2014 had the highest (100%) completeness score for mechanism. PEPID^©^, UptoDate®, DDIC, and DIAM-2014 had a 100% completeness score for management. The overall percentage completeness score was the highest (100%) for DIAM-2014, whereas BNF-76 had a low score (63%) among all the DI resources. The details of completeness scores are presented in Table 5.

**Table 5:** Completeness score for the DI resources studied. PEPID^©^: Portable Electronic Physician Information Database; MDIC: Medscape.com Drug Interaction Checker; DDIC: Drugs.com Drug Interaction Checker; SDI-2010: Stockley’s Drug Interactions, ninth edition, 2010; DIAM-2014: Drug Interactions Analysis & Management-facts and comparisons 2014, ninth edition, 2014; BNF-76: British National Formulary, 76th edition; DI, drug information

Drug information resource	No. of pairs with interaction (X)	Clinical effects [A] (%)	Severity [B] (%)	Mechanism [C] (%)	Management [D] (%)	Overall completeness score [A+B+C+D/X]=Z	Overall % completeness score [Z/4×100]
Micromedex^®^	27	27 (100)	27 (100)	27 (100)	26 (96.2)	3.96	99.00
PEPID^©^	44	44 (100)	44 (100)	37 (84)	44 (100)	3.84	96.02
UpToDate^®^	39	39 (100)	39 (100)	30 (76.9)	39 (100)	3.76	94.23
MDIC	35	35 (100)	35 (100)	28 (80)	29 (82.8)	3.62	90.71
DDIC	41	41 (100)	41 (100)	40 (97.5)	41 (100)	3.97	99.39
SDI-2010	43	43 (100)	43 (100)	33 (76.7)	38 (88.3)	3.65	91.25
DIAM-2014	17	17 (100)	17 (100)	17 (100)	17 (100)	4.00	100
BNF-76	21	21 (100)	16 (76.1)	03 (14.2)	13 (61.9)	2.52	63.00

We observed a poor agreement (value of k less than 0.20) among the DI resources concerning information on all the four components of DDI. The detailed intersource reliability (overall agreement) scores are presented in Table 6.

**Table 6:** Intersource reliability scores for the DI resources studied. DDIs: drug-drug interactions; DI: drug information *p-value less than 0.05 is statistically significant; † k < 0.2 signifies poor agreement

Components of the DDIs	Value of k^†^	95% Confidence interval	P-Value*
Clinical effects	0.188	0.150-0.226	0.000
Severity	0.174	0.145-0.203	0.000
Mechanism	0.122	0.093-0.152	0.000
Management	0.036	0.010-0.061	0.007

## Discussion

We selected 50 drug pairs most commonly used in the general practice irrespective of their therapeutic class or pharmacological actions. In many published studies, the selection of drug pairs was limited to the interactions involving some of the drug classes such as anticoagulants [22,23], dermatological agents [24], antiepileptic drugs [25], anticancer drugs [26], and psychiatric drugs [21,27]. The uniqueness of our drug pair selection was that we also included two drug-vaccine pairs for assessment.

We reviewed three point-of-care databases (Micromedex®, PEPID^©^, and UpToDate®), two online drug interaction checkers (MDIC and DDIC), two textbooks (SDI-2010 and DIAM-2014), and one formulary (BNF-76), which are the most widely used drug information resources in clinical settings in UAE. Among the DI resources reviewed, PEPID^© ^and UpToDate® had entries for all the studied drug pairs, giving them the highest scope score (100%). In comparison, SDI-2010 and BNF-76 had a scope score of 94%. Both of these DI resources had no entry for three of the studied drug pairs. SDI-2010 had no entry for metformin + aspirin, metformin + furosemide, and metformin + HRT, whereas BNF-76 had no entry for ebastine + ketoconazole, thioridazine + duloxetine, and cefpodoxime + pantoprazole combinations.

In a study conducted by Patel and Beckett, Lexicomp® Interactions Module had the highest (97%) scope score compared to our findings where UpToDate® (an integrated DI resource of Lexicomp®) had a scope score of 100%. DIAM-2014 was found to have the lowest (67%) scope score as documented by Patel and Beckett, whereas it was 98% in our assessment for the same DI resource [12].

Patel and Beckett reported the lowest overall completeness score (2 out of 5) for DIAM-2014. In contrast, we observed that DIAM-2014 had the highest completeness score (4, 100%). These differences are mainly due to the different types of drugs selected for the assessment. Additionally, in their study, the drug-dietary supplement interactions were also included for the assessment along with DDIs; however, we focused only on DDIs [12]. DIAM-2014 listed only 17 drug pairs as interacting out of the 50 pairs that we studied. However, the information was complete for all the 17 listed interacting drug pairs in all the components that we studied. This made DIAM-2014 a DI resource with 100% overall completeness score. DIAM-2014 provides detailed information on interacting drug pairs, helping healthcare professionals to decide whether to use the interacting drug pair in their patients after weighing the risk and benefits.

We observed the lowest completeness score (2.62, 63%) for BNF-76. Among 21 interacting drug pairs listed by BNF-76, the information related to only clinical effects was complete (21, 100%) and for other three components, namely, severity (16, 76.1%), mechanism (3, 14.2%), and management (13, 61.9%), had the lowest individual completeness scores, making it a DI resource with the lowest overall completeness score. Being a ready reference resource, BNF-76 can guide whether there is an interaction between drug pairs; however, it has a limitation in guiding how severe the interaction is and how to manage it if it occurs.

Our main objective was to assess the consistency of information among the various DI resources in providing information regarding clinical effects, severity, mechanism, and management. The published research restricted consistency assessment related only to either severity and/or to listing drug interaction pairs [21-30]. In fact, ours is the first study that assessed the consistency of information related to the four aspects of DDIs. Our study’s uniqueness is that we developed and validated the standardized severity rating scale and a numerical score scale to assess the consistency of information across various DI resources (Tables 1, 2; Appendix).

We found that the consistency among DI resources was poor, as assessed by the k score. None of the components studied had a k value more than 0.2, suggesting a statistically poor agreement of consistency among the DI resources studied. We observed inconsistency among the DI resources in listing the interacting drug pairs. None of the assessed DI resources listed all the 50 drug pairs as interacting drug pairs. PEPID^©^ was the only DI resource that listed the highest number (44 out of 50) of interacting drug pairs compared to DIAM-2014 that listed only 17 interacting drug pairs.

Inconsistency was also observed concerning the documentation of clinical effects, levels of severity, mechanism, and management. For example, Micromedex®, PEPID^©^, MDIC, DDIC, and SDI-2010 documented an increased plasma digoxin concentration when digoxin is used along with captopril. Whereas, UpToDate®, DIAM-2014, and BNF-76 documented no interaction between these drug pairs. Digoxin is a narrow therapeutic index drug. When used along with captopril, it increases digoxin plasma concentration and exposes an individual to an increased risk of digoxin toxicity. A healthcare professional referring the DI resources that documented no interaction between this drug pair may expose the patient to increased risk of digoxin toxicity.

Information on the severity of interaction is vital because it is the only deciding factor for the clinical use of interacting drug pairs. In general, if the severity is mild to moderate and the patient condition warrants, the interacting drug pairs can still be used with all the necessary precautions compared to a severe DDI, which must be avoided entirely. We observed a large inconsistency among the DI resources to the severity of the interactions. Micromedex® listed the highest number (n = 18) of interacting drug pairs as severe compared to other DI resources. Only two interacting drug pairs (ketoconazole + simvastatin and glyceryl trinitrate + sildenafil) were categorized as severe in all the DI resources. Surprisingly, the other two interacting drug pairs (metformin + aspirin and thioridazine + duloxetine) categorized as severe in Micromedex® were not at all listed in SDI-2010 and BNF-76, respectively. Many of the severe interacting drug pairs in Micromedex® were categorized as either mild or moderate in other resources. This disagreement puts healthcare professionals at a disadvantage in making the right decision to use such drug pairs in needy patients when the patient condition warrants the use of both medications, especially when there are no alternates available.

In our example of digoxin and captopril, we also observed a similar disparity regarding the severity of the interaction. PEPID^©^ and SDI-2010 documented it as mild, DDIC documented it as moderate, and Micromedex® and MDIC documented it as severe. Concerning the mechanism of interaction, Micromedex® and MDIC documented it as an unknown or unspecified interaction mechanism. PEPID^©^ documented it as a pharmacodynamic drug interaction mechanism, in contrast to DDIC and SDI-2010 that documented it as reduced tubular secretion or impaired renal clearance of digoxin. This information becomes vital when using this drug pair in renally impaired patients at an increased risk of digoxin toxicity. Hence, one must carefully assess the risk and benefits of using this drug combination in such patients.

A wide variation in the management was also observed among the DI resources. The variation ranged from MDIC suggesting to be cautious while using this drug pair, Micromedex®, PEPID^©^, and SDI-2010 advising to monitor the digoxin levels and reduce the doses where possible, to DDIC providing detailed patient education points and involvement of both patients and healthcare professional in identifying and managing the drug interaction. DDIC suggests monitoring of digoxin levels and clinical response by healthcare professional and educating patients on symptoms of digoxin toxicity such as nausea, anorexia, visual disturbances, slow pulse, or irregular heartbeats, and reporting it to their healthcare professionals if they occur.

The differences in documenting information are shared between DI resources. There are various reasons for these differences, and one such reason could be the different sources of evidence. For a practicing healthcare professional, it is crucial to understand these differences to safely use the interacting drug pairs in their patients to prevent any adverse event.

However, in a daily busy schedule, it is practically impossible for prescribers to refer to various DI resources. In addition, sometimes the facilities may have access only to limited DI resources. Therefore, drug information pharmacists are in the right position to overcome these drug-related issues and help the prescribers in the early detection and prevention of potential DDIs, thereby helping prevent adverse events in patients and achieve positive therapeutic benefits.

## Conclusions

In addition to dissimilarity in the listing of interacting drug pairs, a vast disparity was also observed in clinical effects, severity, mechanism, and management of DDIs among DI resources. Lack of reliable information on DDIs, especially on severity and management, complicates the use of interacting drug pairs in at-risk patients by healthcare professionals. Relatively precise information is essential across DI resources to reduce the possibility of adverse clinical outcomes while using interacting drug pairs in clinical practice. We recommend comprehensive preventive and management strategies for identified potential and actual DDIs depending on a uniform scale of assessing severity and clinical effects across various DI resources.

## Appendices

Appendix 1: Development and validation of standardized severity rating scale

The severity rating categories differ from one drug information resource to another. For direct assessment, the observed categories from various drug information resources were classified into three main severity categories: mild, moderate, and major. This categorization was made by understanding the definitions of the categories provided by the drug information resources. By reviewing the drug information resources’ definitions, we redefined the three categories of severity for the standardized severity rating scale. The definitions of various severity categories of standardized severity rating scale are as follows:

**Major:** An interaction that may result in a life-threatening situation, and/or cause a significant hazard, and/or carry a high risk of dangerous outcome where concurrent use is contraindicated.

**Moderate:** An interaction that may result in exacerbation of patients’ condition, and/or cause considerable distress, and/or necessitates an alteration in therapy and/or requires monitoring to reduce the risk.

**Mild:** An interaction that may have limited clinical effects, and/or unlikely to cause incapacitate, and/or carries minimal risk of adverse outcomes, and/or be insignificant, and/or not requires an alteration in therapy.

By definition, the categories such as “contraindicated” and “major” in Micromedex®; “life-threatening” and “significant” in PEPID©; “contraindicated” and “serious-use alternative” in MDIC; “life-threatening; contraindicated” and “significant hazard” in SDI-2010; “avoid-contraindicated” and “major” in DIAM-2014; “severe” in BNF-76; “major” both in DDIC and UpToDate® were considered equivalent to **major **in the standardized severity rating scale.

Whereas categories such as “significant-monitor closely” in MDIC; “consider monitoring” in SDI-2010; “moderate” in DIAM-2014, Micromedex®, PEPID©, BNF-76, DDIC, and UpToDate® were considered equivalent to **moderate** in the standardized severity rating scale.

Similarly, the categories such as “minor” and “minor or non-significant” in PEPID©; “non-significant” in SDI-2010; “mild” in BNF-76; “minor” in Micromedex®, MDIC, DIAM-2014, DDIC, and UpToDate® were considered equivalent to **mild** in the standardized severity rating scale.

Further, for the analysis, all the categories of the drug pairs were numerically coded. The drug pairs that were “not listed” and “non-interacting” as per the drug information resources were coded as 0 and one, respectively. The categories mild, moderate, and major were numerically coded as two, three, and four.

Thus, prepared standardized severity rating scale was pilot tested for 20 drug pairs. At least one drug pair falling in different categories of the drug information resources was selected for pilot testing by the vetting committee. The vetting committee consisted of two doctors from the internal medicine department, a professor from the clinical pharmacy department, and one pharmacist from the community practice and hospital practice.

Each vetting committee member collected and recorded the information related to the severity of the above drug pairs as given in the respective drug information resources. Further, the severity was categorized as per the standardized severity rating scale following the pre-defined definitions. According to the newly developed standardized severity rating scale, the vetting committee members’ consistency in standardizing the severity categories was then estimated utilizing Fleiss Kappa (k) score using ReCal3 0.1 (alpha) web service. As indicated by k score of 1 (very good), the consistency was satisfactory. Thus, standardized scale was used to uniformly classify and code the severity category of all the 50 drug pairs and to assess consistency among the drug information resources concerning severity.

Appendix 2: Development and validation of numerical score scale

A numerical score scale was developed and validated for coding the gathered textual information to ease the consistency assessment concerning clinical effects, mechanism, and management of drug-drug interactions. The numerical score scale ranged between zero and 13. Scores zero and one were given for the drug pairs that were “not listed” and “non-interacting,” respectively. Whereas scores two, three, and four were assigned for rating the severity of drug-drug interactions as mild, moderate, and major, respectively. Scores five through 13 were assigned to code or score the information provided in various drug information resources related to clinical effects, mechanism, and management of the drug-drug interaction. The textual description for the scores are as follows:

**Numerical score five through 12:** If the information in all the eight drug information resources was different from one another.

**Numerical score 13: **If the drug information resource does not provide any information (not mentioned) about the severity or clinical effects, mechanism, or management of interacting drug pairs.

**Identical numerical score: **If the information is identical in more than one drug information resource, all the drug information resources with identical information were given the same numerical score.

Thus, prepared numerical score scale was pilot tested on four drug pairs that the vetting committee selected. Each vetting committee member individually collected and recorded the information related to clinical effects, mechanism, and management of drug-drug interactions for the selected drug pairs as given in the respective drug information resources. Further, the textual information was converted into scores as per the newly developed numerical score scale. The consistency among the vetting committee members in converting the textual information into score codes was then estimated employing k score using ReCal3 0.1 (alpha) web service. As indicated by k score of one (very good), the consistency was satisfactory. Thus, validated numerical score scale was used to convert the textual information into scores for all the 50 drug pairs and to assess the consistency among the drug information resources.
